# Spinal Cord Injury at Birth, Expected Medical and Health Complexity in Chronic Injury Guided Anew by Activity-Based Restorative Therapy: Case Report

**DOI:** 10.3389/fpsyg.2022.800091

**Published:** 2022-04-07

**Authors:** Laura Leon Machado, Kathryn Noonan, Scott Bickel, Goutam Singh, Kyle Brothers, Margaret Calvery, Andrea L. Behrman

**Affiliations:** ^1^UofL Health, Frazier Rehab Institute, Kosair Charities Center for Pediatric NeuroRecovery, Louisville, KY, United States; ^2^Division of Pediatric Pulmonology, Allergy and Immunology, Norton Children’s Hospital, Louisville, KY, United States; ^3^School of Medicine, University of Louisville, Louisville, KY, United States; ^4^Kosair Charities School of Physical Therapy, Spalding University, Louisville, KY, United States; ^5^Norton Children’s Research Institute, Affiliated With the University of Louisville School of Medicine, Louisville, KY, United States; ^6^Norton Children’s Medical Group, Louisville, KY, United States; ^7^Department of Pediatrics, University of Louisville, Louisville, KY, United States; ^8^Department of Neurological Surgery, Kentucky Spinal Cord Injury Research Center, Kosair Charities Center for Pediatric NeuroRecovery, University of Louisville, Louisville, KY, United States

**Keywords:** spinal cord injury, case report, infancy, activity-based restorative therapies, development

## Abstract

As infancy is characterized by rapid physical growth and critical periods of development, disruptions due to illness or disease reveal vulnerability associated with this period. Spinal cord injury (SCI) has devastating consequences at any age, but its onset neonatally, at birth, or within the first year of life multiplies its impact. The immediate physical and physiological consequences are obvious and immense, but the effects on the typical trajectory of development are profound. Activity-based restorative therapies (ABRT) capitalize on activity-dependent plasticity of the neuromuscular system below the lesion and when provided to children with SCI aim to improve the child’s neuromuscular capacity, health and quality of life. This is a report of an infant with a cervical SCI at birth resulting in paralysis of leg and trunk muscles and paresis of arm and hands who was enrolled in an ABRT program at 3 years of age. After 59 sessions of ABRT, the child demonstrated significant improvements in trunk control and arm function, as well as social and emotional development. Despite the chronicity of injury and low expectations for improvement with therapeutic interventions, ABRT had a positive impact on the child’s physical capacity and provided benefits across multiple developmental domains.

## Introduction

Infancy is a rich period of sensorimotor experiences, typically learning through trial and error, that together with maturation and activity-dependent plasticity drive development (Campos et al., 2000; Pape, 2012). Infants who have interruptions to development due to disease, illness, or injury, alter the malleable natural learning process, deviate from this well-known trajectory, and are at risk for maladaptive neuroplasticity (Campos et al., 2000; Pape, 2012; Bhutta et al., 2017; Argetsinger et al., 2020). Spinal cord injury (SCI) presents unique challenges to the health and development of young children when injured under 5 years of age (Schottler et al., 2012), and perhaps even more so when injured at birth. Neonatal or birth associated trauma to the spinal cord may result in death (Abroms et al., 1973; Donn and Faix, 1983; Young et al., 1983; Menticoglou et al., 1995; Mills et al., 2001; Morgan and Newell, 2001; Hedderly et al., 2005; Coulter et al., 2007) or survival (Ruge et al., 1988; Menticoglou et al., 1995; Berck et al., 1998; Kobayashi et al., 2006). Reports detailing the process of rehabilitation and outcomes when a child is injured so very young are limited (Pape, 2012; Felter et al., 2019). While the very low percentage of pediatric-onset SCI (3–5%) relative to the total annual number of individuals with SCI (approximately 10,000) (Nobunaga et al., 1999; Vogel et al., 2004; National Spinal Cord Injury Statistical Center, 2020), the occurrence of SCI *in utero* or at birth is even less. Infant SCI is thus likely commensurate with categorization as an orphan disease. A detailed case report of infants born with SCI *via* medical and rehabilitation history including clinical decision-making thus may be the most appropriate means and highly instructive method to communicate with healthcare providers and advance the care and outcomes of such infants.

After SCI, multiple physiological systems may be compromised during critical periods of development, e.g., respiratory, musculoskeletal, self-directed mobility, and psychosocial (Schottler et al., 2012; Canavese and Dimeglio, 2013). Medical and rehabilitation interventions historically manage physiological systems for health and functional goals after SCI with little expectation of restoration (Schottler et al., 2012), even though developmental plasticity in children has demonstrated better levels of recovery with improvement over years (Pape, 2012). The absence or decrease of trunk control, which should have been fully established between 6 and 12 months (Pin et al., 2018, 2019; Righetto Greco et al., 2020), further complicates development, and access to the critical and typical sensorimotor experiences associated with development. Additionally, these children are at increased risk for scoliosis, hip dysplasia, pneumonia, and development challenges due to the early age at injury (McCarthy et al., 2004; Mulcahey et al., 2013, 2014; Betz and Murray, 2014; Pahys et al., 2014). Furthermore, after 1-year post-injury, rehabilitation expectations for natural recovery diminish. Thus, during the chronic periods post-SCI, only short intervals of therapeutic interventions (1–2 weeks) centered around acquisition of new equipment or developmentally sensitive periods (i.e., early childhood or adolescence) (Vogel et al., 2014) are recommended. Whether the direct effects of injury and its secondary complications on development are mutable by medical or therapeutic interventions is the aim of this exploratory case report. Orthopedic approaches to management of musculoskeletal risks focus on achievement of passive alignment, whether trunk or hips. In contrast, neurotherapeutic approaches, including activity-based restorative therapies (ABRT) (Behrman et al., 2006, 2017; Sadowsky and McDonald, 2009; Roy et al., 2012; Dolbow et al., 2015), aim to activate the neuromuscular system above and below the injury *via* more typical sensorimotor experiences and age-appropriate activities. Two such therapies are activity-based locomotor training (ABLT) (Harkema et al., 2011) and neuromuscular electrical stimulation (NMES) (Collins, 2007; Dean et al., 2007) providing an ensemble of task-specific sensorimotor input to activate the neuromuscular system for posture, standing, stepping, and upper extremity control, also seeking to minimize maladaptive plasticity that comes with largely unintentional deprivation from motor learning and sensorimotor experiences. Based on previous work (Argetsinger et al., 2019), changes in trunk control during ABRT, while seen during the first 20 sessions of an episode of care, continued to show improvements across 60 sessions. This led to the clinical protocol for the neurorecovery program to see pediatric patients for a minimum of 60 sessions during their first episode of care. The outcome measures for the clinical ABRT program are standardized for all patients, they include evidence-based measures that emphasize the kinematics of how the child completes the task versus only the ability to complete [e.g., Pediatric Neuromuscular Recovery Scale and Segmental Assessment of Trunk Control (SATCo)] (Butler et al., 2010; Ardolino et al., 2016; Argetsinger et al., 2019). Standardized outcome measurements were captured at all assessments: initially and every 20 sessions.

We report the case history of a child with a C4-T1 SCI, at birth, seen for usual medical care until 3 years of age and exhibiting expected complications due to SCI in infancy, followed by 3 months of outpatient ABRT that aimed to improve the child’s neuromuscular capacity, health, and quality of life. Comparison and contrast of the approaches and outcomes offer insight into the role of sensorimotor experience even in chronic SCI relative to development. From the standardized bank of outcome measures for the program, relevant outcomes that addressed changes in the trunk, arms, and respiratory functions were reported.

## Case Description

**FIGURE 1:**
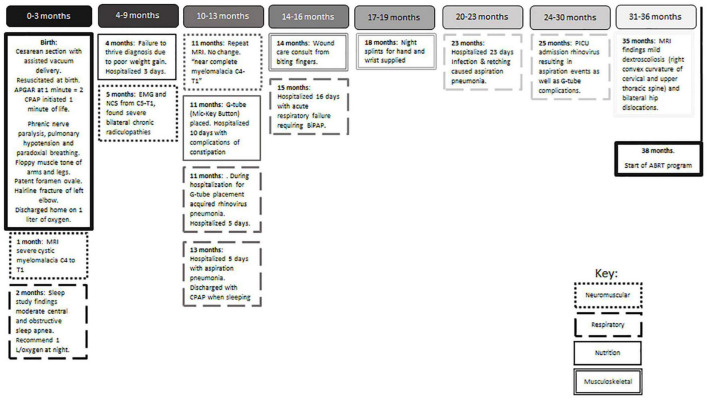
Timeline of medical care required from birth to enrollment in ABRT (38 months).

**FIGURE 2:**
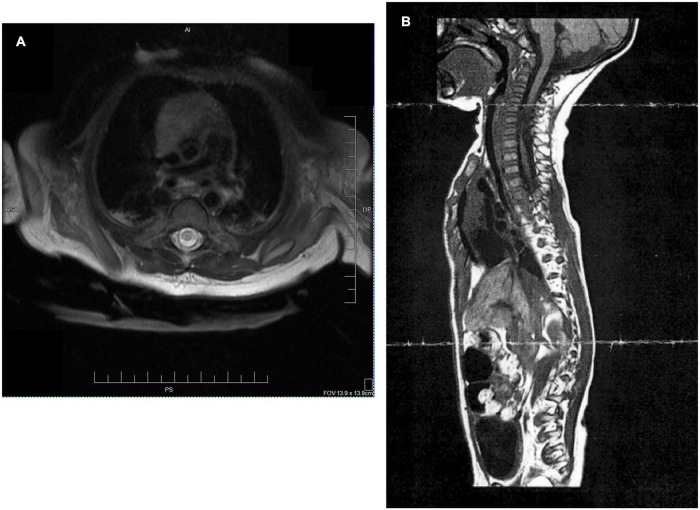
**(A)** Transaxial T2 sequence: MRI of cervical spine revealed cystic myelomalacia involving mid and lower upper thoracic spinal cord between inferior C4 and T1 levels at age 1 month. **(B)** MRI sagittal image at 11 months of age again shows cystic myelomalacia from C4-T1.

An infant, the fourth child of two married parents, was noted to have a low heart rate and floppy presentation upon birth *via* cesarean section with vacuum assist and required respiratory support with CPAP at 1 min of life (Figure 1). As his hospital course continued medical records note “it became clear his neurological status was abnormal and a neonatal neurologist was involved. An extensive workup was undertaken including evaluation for congenital myopathies, mitochondrial/metabolic myopathy, congenital myasthenia gravis, myasthenic syndrome, congenital muscular dystrophy, spinal muscular atrophy, and Prader-Willi syndrome. The patient had a full workup including serum amino acids, urine organic acid, Pompei testing, rapid spinal muscle atrophy testing, CK, LFTs, lactate, pyruvic acid and exome testing; at present this workup has been negative.” Magnetic resonance imaging at one month of age found severe cystic myelomalacia, reflecting previous trauma or spinal cord infarct, from C4-T1 (Figure 2) resulting in tetraplegia and respiratory compromise. According to medical report, the visualized brainstem, cervicomedullary junction, upper cervical and thoracic spine appear within normal limits, vertebral bodies had normal alignment and height, no spinal stenosis and no Chiari I malformation were seen. At 1 month of age, he was discharged home on oxygen support. At his 4-month checkup, he was diagnosed with failure to thrive requiring inpatient admission. Eventually a gastrostomy tube was placed to improve his nutritional status. A repeat MRI at 11 months of age reported similar findings as the first conducted at one month of age. Over the next 3 years, he was repeatedly hospitalized for respiratory concerns including pneumonia and respiratory distress (five admissions). Orthopedic recommendations included wearing of thoracolumbosacral orthosis and supported standing in hip abduction to address bilateral hip dysplasia. Traditional occupational (OT) and physical therapy (PT) emphasized passive musculoskeletal alignment *via* weight-bearing support, splinting and range of motion. These strategies, as well as early intervention services, were initiated at 1 month of age, 2×/week. Per medical record, goals for OT included: tolerance for nighttime splints for hand/tendonesis grasp and elbow extension, and family competence in hand stretching regime. While seen by early intervention services, the child was fitted for a supine to upright stander with significant (beyond shoulder width) hip abduction with the goal of supporting hip socket development.

At enrollment in the ABRT program, age 3 years, he presented in passive mobility (stroller or pushed in a manual wheelchair), he was unable to sit, stand, walk, roll, reach, self-initiate mobility, or self-feed. Unable to sit independently or with upper limb support, his posture was dominated by sacral sitting with trunk kyphotic collapse below ribs and right lateral collapse. Range of motion deficits in bilateral hip adduction (positive Ober’s test) contributed to this slouched alignment. His respiratory pattern comprised of gasping, paradoxical diaphragmatic breathing. Unable to sit without someone supporting him, engagement with his environment was largely limited to observation or toys/food brought to him. His upper limbs were frequently postured and maintained in shoulder abduction and elbow flexion with scapular asymmetry, a non-functional position. Early orthopedic radiologic examination found a flexible spine presenting without scoliosis or fixed kyphosis. Per pulmonologist recommendation, heart rate, oxygen saturation and respiratory rate were collected daily pre- and post-ABRT sessions.

The parents signed an informed consent (IRB# 05.016J, 15.0183 and 19.0137) for placement of their child’s clinical outcome measures in a research data base to be used for future research inquiries and for enrollment in a study of caregivers of children undergoing ABRT. Parent interviews were conducted by a qualitative researcher upon enrollment and discharge. Questions and dialogue focused on prior rehabilitation experience and impact of current restorative therapy experience on the child, family, and parents/caregivers.

## Results

### Intervention

This child completed 59 daily ABRT sessions across 3 months. PT and OT initiated co-treatment due to the child’s age, low endurance, and fatigue. Co-treatments included 1 h of manual-facilitated standing and stepping on a treadmill with the child wearing a trunk and pelvis harness using partial body weight support overhead followed by 30 min of training off the treadmill in sitting or standing for translation of newly developed skills (i.e., ABLT) (Harkema et al., 2011). Harness adjustment for trunk posture was provided *via* circumferential straps that allowed for decreasing amount of support provided as well as arm supports to limit elbow flexion and emphasize movement of arm from shoulder (Table 1). Treadmill stepping was initially challenged as hip adduction range deficit prevented midline alignment for foot placement (Table 1). The child also demonstrated difficulty maintaining head in midline orientation demonstrating lateral tilt or seeking out support from upright straps. As endurance improved, PT and OT sessions separated into two separate sessions (36 sessions). OT initiated use of NMES (Collins, 2007; Dean et al., 2007) on upper extremities and trunk during reaching overhead and weight bearing through arms for scapular stabilization while PT continued ABLT. The child was re-assessed every 20 sessions.

**TABLE 1:** Outcomes in locomotor training treadmill environment.

Outcomes–locomotor training treadmill environment		
	**Initial evaluation**	**Discharge evaluations**
Pulmonary measures–respiratory rate	Pre-stepping = 41 bpm Post-stepping = 39 bpm	Pre-stepping = 38 bpm Post-stepping = 28 bpm
Pulmonary measures–pulse oximetry	Pre-stepping = 97% Post-stepping = 97%	Pre-stepping = 98% Post-stepping = 98%
Body weight support	65%	48%
Stepping speed (age appropriate speed ≥1.5 mph)	0.5 mph	1.9 mph
Trunk support *via* harness circumferential band placement	Two trunk straps support axillae	Single trunk strap support low ribs
	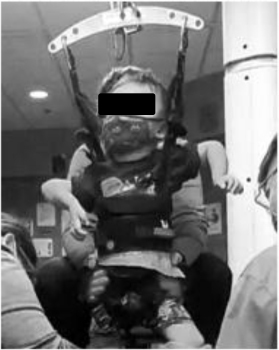	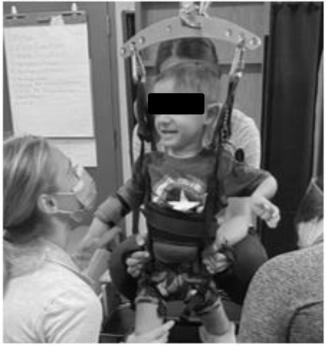
Steps width–impact of hip adduction range limitation	Wide width toward edge of 14′′ treadmill belt. Exceeds shoulder width	Decreased stepping width. More toward midline of treadmill belt. Appropriate shoulder width
	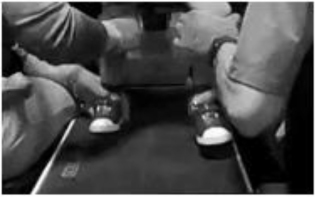	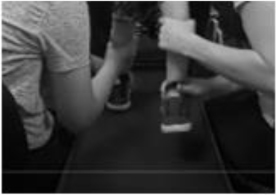

*The child demonstrated stable pulmonary measures including respiratory rate (bpm, breaths per minute) and pulse oximetry percentage. From initial evaluation to discharge, the child’s increasing neuromuscular capacity allowed for decreases in percentage of body weight support and increases in facilitated stepping speed while still maintaining appropriate kinematics at trunk, pelvis, and legs. The ability to remove circumferential trunk straps due to increases in trunk control and more age-appropriate step width were also seen.*

Clinical decisions were continually made during sessions with a focus on promotion of postural symmetry at trunk and head, arm swing and weight-bearing through legs. Once the child was able to maintain midline head alignment, trunk support was decreased *via* removal of circumferential support straps to further challenge his postural control. While stepping, an emphasis was placed on arm swing, with initial facilitation on right moving toward bilaterally self-initiated arm swing by discharge. The child was engaged in imaginary play with a staff member during stepping bouts to enhance motivation and engagement of movement. During standing activities, overhead reach occurred with extended arms to emphasize trunk extension and upper extremity use promoting postural control and play.

### Respiratory Function

Per pulmonologist recommendation, oxygen saturation and respiratory rate were measured in standing before and after stepping on treadmill. Periodically these measures were provided for review by the pulmonologist. Over the course of therapy these measures remained stable (Table 1).

During baseline airway pressure assessment (Singh et al., 2018), the child was positioned in supported sitting *via* a backrest. He was unable to generate or perform maximal inspiratory airway pressure (PImax), whereas baseline maximal expiratory airway pressure (PEmax) was 9 ± 0.4 cm of H20. PEmax increased from 9 ± 0.4 cm of H20 to 13 ± 0.4 cm of H20 post intervention. Post intervention, the child was able to generate a PImax of −16 ± 3 cm of H20. Such tests are typically performed as early as 4–5 years of age due to the need to understand and follow instructions and to give maximal effort during testing. The tests were attempted with this 3-year-old to provide a baseline and will be repeated as the child ages with future episodes of care.

### Trunk Control

**FIGURE 3:**
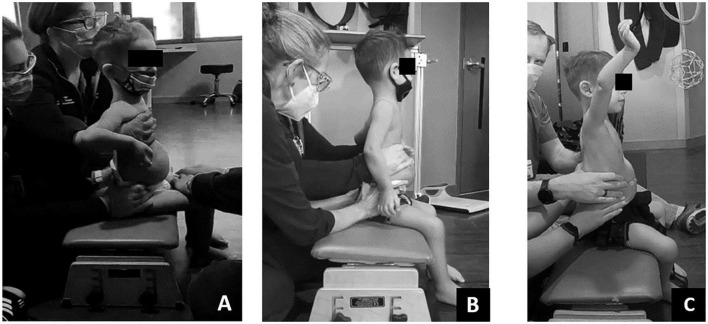
Changes in Segmental Assessment of Trunk Control (SATCo) across time. **(A)** Initial evaluation with neutral pelvis and support at axillae. **(B)** At 20 sessions of ABRT neutral pelvis and support at inferior scapulae and trunk controlled above. **(C)** Upon discharge evaluation with neutral pelvis external support at low ribs and trunk control above.

The SATCo (Butler et al., 2010) was used as it was developed to evaluate trunk control in children who cannot sit independently and is responsive to change in children with SCI (Argetsinger et al., 2019). The child’s initial SATCo score was 5/20, meaning with support at the axillae and pelvis to establish a neutral pelvis alignment and firm base, he was able to maintain upper thoracic control above the support with his head in midline. At discharge his SATCo score was 10/20, meaning the child could now appropriately maintain alignment of his trunk above external support at low ribs (i.e., lower thoracic control) with the pelvis supported in neutral alignment (Figure 3).

**FIGURE 4:**
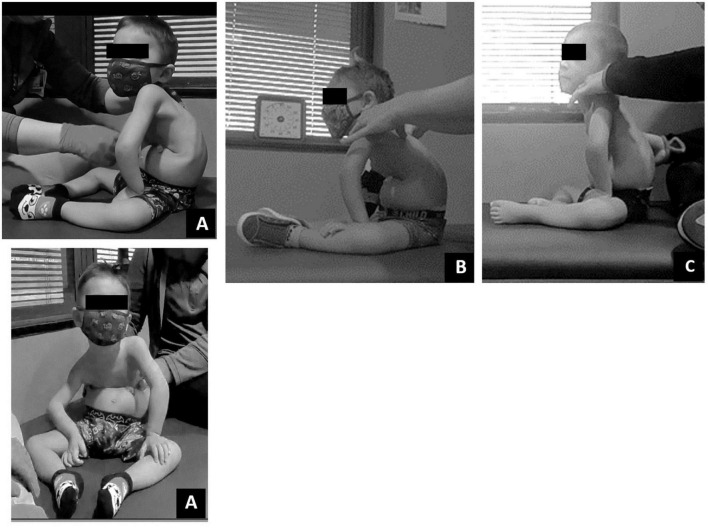
Dependent sitting posture change over time. **(A)** Initial evaluation – lateral and frontal view. Sacral sitting with lower extremity abduction and external rotation, sharp flexion in trunk at low ribs with kyphotic upper trunk. Right lateral trunk collapse. **(B)** Twenty sessions evaluation. Pelvic alignment demonstrates less sacral sitting with change toward vertical upright alignment. Trunk continued to demonstrate forward lean/kyphotic posture. **(C)** Discharge evaluation. Trunk alignment less kyphotic/more upright toward vertical position while pelvis maintained more vertical alignment toward neutral pelvic position.

**FIGURE 5:**
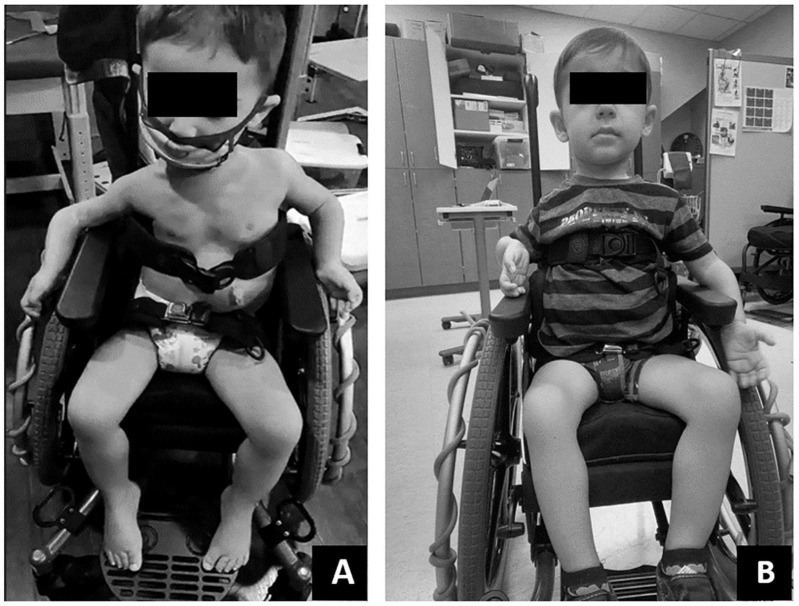
**(A)** Initially this child demonstrated poor postural positioning while in his manual wheelchair including lack of contact with foot plate, sacral sitting, hip abduction, lateral collapse at the trunk and laterally tilted head position. **(B)** With gains in postural control at the trunk and pelvis and minor modifications to his manual chairs pelvic positioning belt and foot plate, this child demonstrated improved trunk symmetry, upright pelvic alignment and weight bearing through feet.

Initially this child required support from someone to maintain sitting in ring, long or short sitting. As the episode of care progressed, trunk and pelvis posture in sitting improved from full sacral sitting toward upright alignment of the pelvis and trunk. At discharge, the child’s trunk capacity changed with the novel ability to prop sit in ring sitting, with his arms on his legs, without support for 20 s (Figure 4). Lastly, changes in his trunk capacity and modifications to his manual wheelchair allowed for a more upright posture at pelvis and trunk (Figure 5).

### Functional Arm Use

**FIGURE 6:**
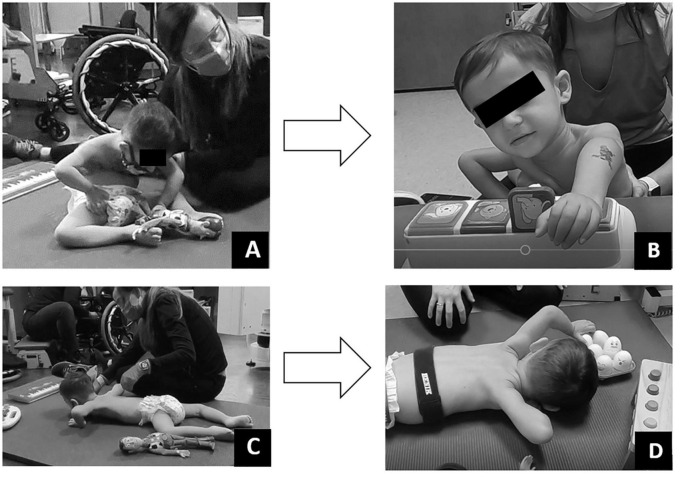
Play assessment. **(A)** Initial evaluation in supported sitting child could minimally touch toy but was unable to manipulate. **(B)** Discharge evaluation in supported sitting child could touch and manipulate toys within his reach. **(C)** Initial evaluation in prone child could not move arms forward to reach and engage. **(D)** Discharge evaluation in prone, child able to reach forward to engage and manipulate toys within his reach.

With neutral pelvis and aligned trunk below low ribs, the child could now engage his upper limbs for feeding and play at a tabletop, and actively point and reach overhead while maintaining elbow extension and neutral shoulder position. With facilitation of gross grasp, the child demonstrated the ability to maintain grasp on a variety of objects engaging in age-appropriate play activities reaching forward and overhead (Figure 6). The child improved left upper extremity gross manual dexterity independently grasping blocks with the lateral aspect of his first digit and pad of his second digit. Improved arm and trunk control allowed successful trialing of a power wheelchair with left hand joystick control for independent mobility.

### Play/Engagement

Play was assessed with a modified play assessment (Kelly-Vance and Ryalls, 2005). At initial evaluation, the child required assistance to attain sitting and prone positioning. Once in sitting, he demonstrated poor postural control requiring support to maintain an upright sitting position. Even in supported sitting, the patient demonstrated a collapsed trunk with only the ability to touch objects directly aligned with hands, without interaction or manipulation of the toy. Once in the prone position, the patient maintained his hands in contact with the floor at the level of axillae (Figure 6). Pre-ABRT assessments revealed patient’s inability to crawl or roll, restricting access to and inhibiting exploration of the environment. Even when placed in the environment, the patient’s inability to extend arms for reach severely restricted his ability to engage within his environment.

Post-ABRT, play assessment revealed patient’s ability to extend arm (left > right) and spontaneously manipulate, explore, and enjoy toys (Figure 6). Upon discharge and interview, the mother reported “It’s really great, see his arm movements, being able to be more independent with his arms. Because we want to see him moving the wheelchair on his own… We want to be able to see him be independent… And I want to be able to see him go to where he wants; go rummage through whatever he wants to get into” in contrast to us “putting him here and there.”

During the episode of care, the parents reported now being able to hold their child upright on their hip with one arm vs. cradling him in an infant position, allowing for novel exposure and with consistent potential for interaction with individuals, items, animals, and toys at adult eye level.

### Social Emotional

The parents provided repeated daily “tidbits” and statements on interview reflecting significant social emotional changes previously inaccessible for the patient due to his severe physical restriction. The father stated, “…he’s really a different kid… he was always quiet, it’s just he seems (now) like more of a kid, that like he wants to joke around. Say funny stuff and do something, wants to go over here, wants to see that he wants to go to the park…. He’s just being more of a kid, you know what I mean, and not necessarily somebody with an injury, that’s just there.”

In addition to play and exploration, essential for cognitive and emotional growth, the patient began to self-feed and “get his cup to his mouth better” in contrast to “me constantly saying here, you want your drink, (he) drinks when he wants it. I’m not shoving it in his face when he doesn’t need it.”

The mother reported the patient developed increasing likes and dislikes with the ability to acquire food and drink: “he’s even more specific too; no I want to eat this. No I don’t like that. I do want to eat this.” She additionally stated, “…his vocabulary is even bigger.” Mother insightfully reports, “I think it’s (ABRT) helping him mentally too, like to explore and like do things and it’s just opening up that even more. The more he talks and says the things that he wants is giving him the ability to have even more verbal skills of this and this and this.”

### Parental Reports

Parent reflections noted the rapid acceleration of progress during ABRT, that “exceeded their expectations,” reflecting the gains acquired by increased action and interactions with the sensorimotor environment. Parent noted a specific incident when child asked, “What’s that?” referring to the new handicapped sign hanging in their vehicle. The parent reported, “We have that because you can’t walk.” The child replied “I walk. I walk on the treadmill.” Upon discharge the mother commented about her child’s improved “trunk control” and associated “strength allowing him to (now) blow bubbles and to sit in a highchair on his own with everyone sitting around the dinner table together.”

## Discussion

Even in a child with chronic SCI and paralysis of legs, trunk, and paresis of arms, the sensorimotor experiences afforded the child *via* ABRT made a meaningful difference. Prior therapy focused on static orthopedic positioning through braces and splints, however he continued to present with asymmetric posture and kyphosis with a history of numerous respiratory illnesses. From birth to 3 years old, he was a passive recipient of his environment, relying on caregivers and medical providers for mobility, play, postural support, and activities of daily living. New sensorimotor experiences provided during the ABRT program emphasized symmetrical supported, dynamic experiences (e.g., stepping, arm swing, upright standing, and upper limb position for play while upright). The child’s improved endurance and activation of trunk alignment allowed for engagement of his arms and literally expanded his accessible world for play, while concurrent improvements in respiratory function occurred consistent with those observed in adults with SCI undergoing ABLT (Terson de Paleville et al., 2013; Hormigo et al., 2017; Sunshine et al., 2020). Neural linkages between respiratory and locomotor systems may also promote activation of trunk muscles within the context of upright stepping during ABLT (Sunshine et al., 2020). The current report and the two publications presenting cases by Felter et al. (2019) and Pape (2012) follow children injured as a neonate: two during birth and the current of unknown etiology across time outlining their therapeutic interventions and outcomes. Pape (2012) offers a brief case report using NMES, a form of sensory stimulation intended to modulate the intrinsic excitability of the spinal cord, in a 3-year-old with chronic SCI due to a C2-3 level injury with ventilator dependency due to birth delivery with a mid-forceps rotation. Details of the intervention frequency and methodology are sparse to absent; however, the child was treated across a 3-year period making steady gains in sitting posture and ability to walk. Both the case reported by Pape (2012) and the current case support the use of NMES to promote neural activation below the level of lesion with positive results in children with chronic SCI. This is important as plasticity in response to an intervention in the chronic state of SCI is typically thought to be highly unlikely and thus those children are often not considered for further neurotherapeutic approaches. Key differences between the Felter case and the current report include the intensity of intervention, duration of intervention and chronicity of injury at onset of ABRT. Felter reports a case beginning ABRT at 11 months of age with a high frequency (6×/week) for one month duration. From age 1 to 3.5 years, frequency diminished to 1×/week for a total of 120 PT and 142 OT visits. In contrast, this report outlines a child, 3 years of age with a chronic injury since birth, receiving high intensity ABRT (5×/week) across a short duration (60 sessions across 3 months). This case also highlights two very specific interventions (ABLT and NMES) within the context of activities (play and practical) to activate the neuromuscular system below the level of the lesion, opposed to Felter where numerous interventions including restorative and compensatory were used. Lastly, differences are seen in the reported outcome measures, whereas Felter reports on use of pediatric scales [Gross Motor Functional Measure-88 and Physical Abilities and Mobility Scale (Russell et al., 2002; Trovato et al., 2013)] used to describe general skills and functions, the current case reports on scales focusing on neuromuscular capacity performed within functional tasks, well complementing the restorative based program. Additionally, the current case includes physiological assessments measuring respiratory capacity. Results of this child’s participation in the daily, ABRT program reveal significant changes across targeted developmental domains as well as spontaneous, unexpected, and non-targeted changes across all developmental domains. Consistent with developmental theory (Piaget et al., 1969; Kopp and Shaperman, 1973; Gibson, 1988; Campos et al., 2000; Soska et al., 2010; Adolph and Robinson, 2015; Adolph and Hoch, 2019), physical, cognitive, adaptive, social, and emotional growth are reciprocal and interconnected. Significant restriction in one domain is expected to impact functioning across other domains. In turn, significant progress in one domain, fuels the developmental system across other domains. In sum, the cognitive development of children requires actions and interactions that translate into growth of mental operations and abilities. Children in the Sensorimotor Stage (birth to 2 years) (Piaget et al., 1969) are in the earliest stage of cognitive development. Infants and toddlers experience the world through basic senses, motor responses, and reflexes. Infants and toddlers acquire knowledge *via* sensory and motor experiences including developing more complex movement patterns and the manipulation of objects. Active interaction with the environment provides the essential ripe learning environment for new learning.

Paralysis imposes immobility and restricts an infant and toddler’s (misnomer for children who are paralyzed and never experience toddling or walking) sensorimotor experiences and exploration. The upright load bearing position during facilitated standing and stepping of ABLT not only had a physiological effect on postural control and arm function but also afforded opportunity for social engagement, interactions with objects and the environment and discovery. The child’s own self-image changed during the course of ABRT and reflected a new “capacity,” i.e., “I walk. I walk on the treadmill.” New neuromuscular capacities (e.g., sitting in a highchair) expanded the child’s engagement and participation in something as not only everyday but also as critical as the shared family experiences of sitting together at dinner. ABRT enabled this 3-year-old child, with severe paralysis, and initially limited physical, social, and mental opportunities to explore and discover, greater capacity to play and explore and thus meaningfully enhanced his development and quality of life.

In contrast to adults with SCI who have reached skeletal and developmental maturity, when injured at such an early age, children with SCI may require ongoing therapeutic support during periods of musculoskeletal growth and human development. This is consistent with the perspective that motor development is “embodied and embedded” and thus a consequence of the body’s characteristics, e.g., changing biomechanics, strength, musculoskeletal growth and of the physical world in which the body exists (Adolph and Hoch, 2019). Thus, changing this child’s physical world from seated with a kyphotic posture in a wheelchair to standing, supported upright and stepping on a treadmill and to sitting upright afforded him the opportunity to use his body more effectively and to develop new motor skills. Mechanisms of activity-dependent plasticity, available to typically developing children, are also available to children injured as infants with SCI. Thus, the environment presented to the injured body for daily experience is critical to the child’s development as it is to children without SCI. Even with the profound changes seen across this child’s short episode of care, he remains at risk for musculoskeletal maldevelopment (e.g., scoliosis, range of motion deficits and hip dysplasia). Therapies targeting trunk control remain relevant to improvement of sitting posture, engagement of arms in activities, and independent power mobility. While ABRT afforded him improvements toward recovery, he will continue to benefit from intense episodes of care with his changing body to support growth and musculoskeletal alignment, promote health and expand his capacity through activation of the trunk, pelvis and arms to engage in his environment.

## Limitations and Conclusion

The findings of this single case report, though limited to this individual, demonstrate neuroplastic potential within a child with a chronic, neonatal SCI when exposed to a permissive therapeutic environment using intense repeated practice to capitalize on activity-dependent plasticity. While a definitive etiology is lacking, the MRI findings and physical presentation were used to guide clinical decision making in both subsequent rehabilitation approaches: passive musculoskeletal alignment and ABRT. These two approaches differ in intent and outcome. The passive alignment approach aims for the infant to use muscular capacity which is available above the lesion and maintain skeletal alignment below the lesion. In contrast, ABRT aims to activate the neuromuscular system both above and uniquely below the lesion and improve the long-term neuromuscular capacity of the child for daily activities. The use of a standardized intervention and outcomes adds to the validity and repeatability of this therapeutic intervention in future cases. In this case, complicated both by developmental delay due to paralysis and ongoing developmental maturation, even a brief 3-month episode of care of intense ABRT, positively altered this child’s capacity and developmental trajectory. While this case is certainly not confirmatory for the implementation of ABRT in this population, it does reinforce the potential even in a child with a chronic injury from birth and provide case evidence for future clinical decision making.

## Data Availability Statement

The original contributions presented in the study are included in the article/Supplementary Material, further inquiries can be directed to the corresponding author.

## Ethics Statement

The studies involving human participants were reviewed and approved by the University of Louisville Institutional Review Board. Written informed consent to participate in this study was provided by the participants’ legal guardian/next of kin. Written informed consent was obtained from the individual(s), and minor(s)’ legal guardian/next of kin, for the publication of any potentially identifiable images or data included in this article.

## Author Contributions

LL oversaw the physical therapy clinical data collection and drafted the manuscript. KN oversaw the occupational therapy clinical data collection and provided manuscript edits. SB oversaw pulmonary care for this child and provided manuscript edits. GS completed respiratory outcome measures and provided manuscript edits. KB assisted with caregiver interviews and related input for the manuscript. MC oversaw developmental psychology care and provided related input for manuscript. AB oversaw all clinical and research interventions for this child and drafted and edited the manuscript. All authors contributed to the article and approved the submitted version.

## Conflict of Interest

AB is volunteer president of NeuroRecovery Learning, Inc., a non-profit organization that works to expedite the translation of new scientific findings for the care of those living with spinal cord injury. She is also a co-author of Locomotor Training Principles and Practice (2011), receiving royalties from Oxford University Press. The University of Louisville licenses a specialized pediatric treadmill and body-weight support system and harness co-developed by AB. The remaining authors declare that the research was conducted in the absence of any commercial or financial relationships that could be construed as a potential conflict of interest.

## Publisher’s Note

All claims expressed in this article are solely those of the authors and do not necessarily represent those of their affiliated organizations, or those of the publisher, the editors and the reviewers. Any product that may be evaluated in this article, or claim that may be made by its manufacturer, is not guaranteed or endorsed by the publisher.
