# Supplementary material for: Spinal Cord Injury at Birth, Expected Medical and Health Complexity in Chronic Injury Guided Anew by Activity-Based Restorative Therapy: Case Report
Source: Front Psychol. 2022 Apr 7;13:800091. doi: 10.3389/fpsyg.2022.800091 (PMC9021874; doi:10.3389/fpsyg.2022.800091)
Supplement: Supplementary file 1 [file Table_1.DOCX]

**Relevant Excerpts from Discharge Caregiver Interview**

Mother: “And also, with his age with him being older and talking, he's been talking pretty well before we got here, but I can even tell his vocabulary is even bigger. And he's right away of like, oh, I'm hungry or I'm thirsty. Can I get my milk? He's even more specific too; oh, I want to eat this. No, I don't like that. I do want to eat this. That's helped a lot too, just him verbalizing what it is he likes and doesn't like. And, and letting us know even not just when it comes to food too like, oh I want to get in my stander. I want to go in my wheelchair. I want to play with this. I want to do this. At least him giving us a heads up of what he wants to do gives us even more of an ability to put him in the areas that he wants to be independent too. Like, oh you want to get in your wheelchair? Here you go. And let him do his own thing with his own toys on his tray or in that area that he wants to be in. So, I think it's helping him mentally too, like to explore and like do things and it's just opening up that even more, the more he talks and says the things that he wants is giving him the ability to have even more verbal skills of this and this and this. And yeah, those physical abilities to play with the things that he wants to too is really encouraging. Because, I think the more he does it, or when he even surprises himself and does things that he may not have realized he was able to do on his own, I think pushes him even further to want to do even more.”

Mother: “Let’s see. I think sometimes he'll tell us, like when we put toys in front of him on his tray and he has certain things right there, and he'll ask for like Thor, he has this little Thor doll and he has like a Thor hammer and he'll say, I want that hammer, I want, you know. He's really used to just asking us for everything, you know. I want the hammer, I want this, I want that, please can I have this. And we'll tell him you get it, you know. No, you grab it. And at first, he'll say like I can't, I can't, I can't. And then you'll see him keep trying even trying, you know, he’ll try to pick it up and he can't, he can’t, he’ll keep saying he can't, but we're like, well, I'm not getting it then. And we just tell him we won't. And he'll eventually get it and I'll say see, you did it. Oh yeah. Look, I got it. Like, he'll say things like that, like I did it, you know, he'll be surprised. Or sometimes we'll pretend like, kind of brush it off, well I'm not going to get it. And you just kind of act like you're busy doing whatever. You know, when really, you're sitting there testing him more, like, kind of watching, like you couldn't get it, is he going to get it? But don't let him know you know, make him think like oh it's nothing, whatever. Because the more you like push or say okay, like sometimes the more encouraging you are, the less like he's more like I can't. See, I can’t, and he just kind of messes around. But if you kind of pretend like you're not paying attention, he's going to try more on his own. And then all of a sudden, he’ll be like, look Mom look, look, you know, to tell you. But there have been moments even on his own, where he's messing around and he grabbed something, because he's close enough to it, and I'll say, look, look what I have, look what I got. And at that moment, like, that's when you know, like oh, he's, he's happy that he just grabbed something on his own, you know. Like he did that independently, like nobody asked him to or even if he thinks he might get in trouble for grabbing something that he shouldn't have or knocking something over, it's still actually good to see like he did that on his own. He did something that other kids are always getting into stuff and getting trouble and being told no, don't touch that. Like, he needs that too, so it's good to see when he does that.”
